# Evaluation of Changes in Salivary pH after Intake of Different Eatables and Beverages in Children at Different Time Intervals

**DOI:** 10.5005/jp-journals-10005-1507

**Published:** 2018-06-01

**Authors:** Ankit Pachori, Haalaswamy Kambalimath, Satish Maran, Babita Niranjan, Garima Bhambhani, Garima Malhotra

**Affiliations:** 1 Postgraduate Student (Final Year), Department of Pedodontics and Preventive Dentistry, Rishiraj College of Dental Sciences and Research Centre, Bhopal Madhya Pradesh, India; 2 Professor and Head, Department of Pedodontics and Preventive Dentistry, Rishiraj College of Dental Sciences and Research Centre, Bhopal Madhya Pradesh, India; 3 Senior Lecturer, Department of Pedodontics and Preventive Dentistry, Rishiraj College of Dental Sciences and Research Centre, Bhopal Madhya Pradesh, India; 4 Senior Lecturer, Department of Pedodontics and Preventive Dentistry, Rishiraj College of Dental Sciences and Research Centre, Bhopal Madhya Pradesh, India; 5 Senior Lecturer, Department of Public Health Dentistry, Peoples College of Dental Sciences, Bhopal, Madhya Pradesh, India; 6 Senior Lecturer, Department of Pedodontics and Preventive Dentistry, Maharana Pratap College of Dental Sciences, Gwalior, Madhya Pradesh India

**Keywords:** Buffer capacity, Erosion, Salivary pH.

## Abstract

**Introduction:**

Dissolution of the enamel in the form of erosion has increased due to shift in eating habits toward carbonated drinks like soft drink and chocolates.

**Aim:**

The purpose of this study was to evaluate changes in the salivary pH after intake of different eatables and beverages in children at different time intervals.

**Study design:**

Using standard salivary tests, this study was conducted as a case-control study. Fifty children between ages of 8 and 12 years having strict vegetarian diet were included in the study.

**Materials and methods:**

Oral prophylaxis was done and salivary buffer capacity for stimulated saliva was measured. Before the saliva collection, each of the children was informed not to eat or drink anything for up to 2 hours just before the appointment.

**Results:**

On pairwise evaluation, all the differences were found to be significant statistically except between baseline *vs* 30 minutes. Maximum mean difference was calculated and observed between baseline and immediately after intake of fruit juice and immediate after 30 minutes, 0.953 and 0.963 respectively, while minimum difference was calculated and observed between baseline and 30 minutes after intake of fruit juice (0.010).

**Conclusion:**

Maximum drop was seen in cold drink when compared with fruit juice. The maximum pH increase was observed in cream biscuits with 7.63 ± 0.20. It was observed that in all the groups, the pH gradually got back to near normal levels due to the buffering mechanism of saliva.

**How to cite this article:** Pachori A, Kambalimath H, Maran S, Niranjan B, Bhambhani G, Malhotra G. Evaluation of Changes in Salivary pH after Intake of Different Eatables and Beverages in Children at Different Time Intervals. Int J Clin Pediatr Dent 2018;11(3):177-182.

## INTRODUCTION

The dissolution of the enamel leads to two distinct types of lesions, a carious lesion or erosion. By definition, a carious lesion occurs as a result of acid formed by bacterial degradation of sugars, while erosion results from chemical dissolution caused by acid of any other origin,^1^ while the etiologic factors for dental erosion are acids of intrinsic and extrinsic origin mainly. However, in case of dietary substances, the pH alone cannot be predictive of potential of any acidic food stuff or beverages to cause erosion, as other chemical factors like adhesion and chelating properties, pKa values, calcium, phosphate, and fluoride content, behavioral factors like eating and drinking habits, excessive consumption of acids, lifestyle and biological factors like composition of saliva, flow rate, buffering capacity, dental and soft tissue anatomy, pellicle formation, dental and soft tissue anatomy, tooth composition, etc., modify the erosive process.^2^

Among many other causes of dental caries, diet has long been acknowledged as a major cause.^3^ Osborn et al compared the amount of tooth demineralization by incubating the foods in saliva to explain the different caries activity in users of coarse and refined cereals and sugars. Many varieties of food available in the market and foods which contain sucrose are considered to be the major constituent in the initiation of caries.^4^

The amount of enamel dissolution by salivary fermentation increases as the sucrose content increases, causing increase in the acidity of microbial origin and leading to potential caries formation. Also, cariogenic potential of food can be determined by various factors, such as amount of acid formed on fermentation in bacteria, salivary retention time, and the conditions under which eatables are consumed.^1^ A high incidence of lesions had been found in children and teenagers, reflecting a high intake of acidic food and beverages.^5^ Most common source of dietary sugars includes cold drinks, confectionaries, biscuits, and breakfast cereals. Recent data from the NDN survey (National Diet and Nutrition survey), Britain, have shown that soft drinks make up more than 25% and confectionary more than 20% of the total nonmilk extrinsic sugar consumed by children.^3^ Commercially available fruit juices and soft drinks contain organic acids and sugar substitutes as natural ingredients, which can not only lead to health problems in children but also damage dental hard tissues.^6^ The caustic effect of fruit juices has been recognized for a long time as evident in the studies of Darby (1892) and Miller (1907) who concluded that tooth decalcification occurred due to excessive consumption of fruit juice.^7^ According to Harnandez et al, Drake and Eisele (1999), Gokmenet et al, the variety, the geographical origin, the time of harvesting, and treatment and the processing methods of fruits all influence the composition of the fruit juices, and thus the amount of sugars and organic acids.^8^ Now the world has a passion for chocolate with average 16 to 21 lb annually in various parts of the world. Individual studies were done on the outcome of chocolate consumption on plaque pH and flow rate of the saliva but none of them were done on salivary pH.^9^

As globalization in market strategies for production and distribution has a significant worldwide impact on dietary excess leading to various chronic diseases, there is a significant effect on the oral health of the young individuals. Along with high rise in consumption of cold drinks, packaged fruit juices and chocolates, consumption of quick snacks like potato chip wafers and cream biscuits has led to increase in concern of the health policy makers. Hence, this study was conducted to assess the acidogenic potential of cold drink, packaged fruit juice, chocolate, potato chips, and cream biscuits, and evaluate the buffer capacity and its relationship with the acidogenicity of these products after consumption.

## MATERIALS AND METHODS

Fifty children between the age of 8 and 12 years visiting the Outpatient Department of Paedodontics and Preventive Dentistry, who fulfilled inclusion criteria were included in the study. Ethical clearance was acquired from the ethical committee and written informed consent was taken from the parent of each patient. The selected children after exclusion and inclusion criteria were assessed on the first day of study. Procedure was explained to them, oral prophylaxis was done, and salivary buffer capacity for stimulated saliva was measured. All salivary tests were performed by only one person on whole saliva. Before the saliva collection, each of the child was informed not to eat or drink anything for up to 2 hours just before the appointment. Parent of each subject was given reasons for the tests, with simple explanation before collecting saliva samples.

### Quantification of Salivary Buffer Capacity for Stimulated Saliva

Quantification of salivary buffer capacity for stimulated saliva was carried out using GC Saliva-Check BUFFER kit (GC India Dental Pvt. Ltd.) as per the manufacturer’s instructions. For measurement, the foil package was opened and a buffer test strip was purged and with the test side up placed onto an absorbent paper. Saliva in sufficient amount was drawn from the collection cup using a pipette provided with kit and one drop of saliva was released over each of the three well-marked test pads on the strip. To soak up excess saliva on the absorbent paper immediately, the strip was turned 90° to the surface of paper. By doing this, excess saliva was prevented to swell on the test pad which could possibly affect the test result accuracy. The test pads began changing color immediately and the final result was calculated after 2 minutes by adding points based on the observed final color for each of the pads on the strip. Conversion table for test pad color was provided in the instruction manual provided by the manufacturer (Tables 1 and 2).

### Measurement of Salivary pH

The salivary pH of both unstimulated and stimulated saliva was estimated directly using a MAX bench type ME-62 Microprocessor pH/mV/Temp. Unstimulated pH was estimated during the same time of the day. The pH meter was calibrated each day prior to measurement with buffering solution having pH 7 and 4 respectively, for an accurate reading. For collection of unstimulated saliva, all children were asked to sit comfortably on a chair, with their head bent forward for easy collection of sample in test tubes. After baseline score was documented, eatables and beverages were tested on all children. For five subsequent days, different eatables and beverages were given to children. Test eatables and drinks were given to children in premeasured cups and were asked to drink beverages from a glass without using a straw. They were asked to sip each beverage and eat as they usually do at home. After consumption of different eatables and beverages, immediately salivary pH was measured. Salivary pH was measured at an interval of 5, 10, 15, and 30 minutes. At 2nd, 3rd, 4th and 5th day, eatables and beverages given were potato chips, packaged fruit juice, cold drink, and chocolates respectively.

**Table 1:** **Table 1:** Conversion table: Test pad color after 2 minutes

Green			4 points	
Green/blue			3 points*	
Blue			2 points	
Red/blue			1 point*	
Red			0 points	

*When any color combination indicates unclear result, use intermediate scores

**Table 2:** **Table 2:** Interpretation of the result

*Combined total*			*Buffering ability of saliva*	
0-5			Very low	
6-9			Low	
10-12			Normal/high	

The whole procedure was supervised and performed by a single investigator. To measure salivary pH, saliva was accumulated in different test tubes for each interval. The pH-sensitive glass electrode (MAX ME-62 [Max Electronics India]) was immersed in sample for the reading. In between readings, cleaning of electrode with a stream of distilled water was carried out which was then dipped in a standard solution having pH 70, to ensure a stable reading and salivary pH was immediately measured after collection.

The data obtained were compiled systematically. Statistical analysis was done using Statistical Package for the Social Science (Version 20; Chicago Inc., USA). Data comparison was done by applying specific statistical tests to find out statistical significance of the comparisons. Quantitative variables were compared using mean values and qualitative variables using proportions. Analysis of variance (ANOVA) test was used to compare within-group and between-group (for intergroup comparison) variances in the study.

## RESULTS

### Intergroup Comparison

**Graph 1:**
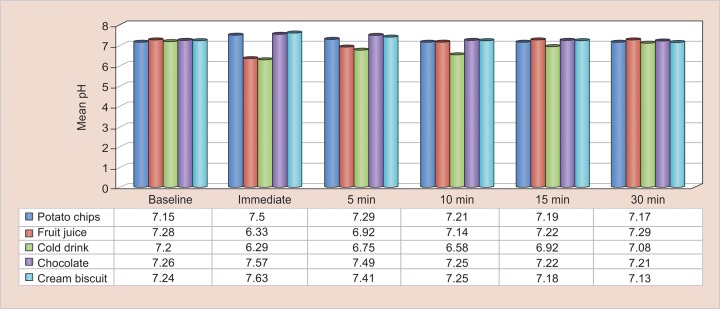
Comparative bar graph for mean pH values in different groups at different intervals

At baseline, mean salivary pH levels in different groups ranged from 7.15 to 7.28. It was found to be highest for fruit juice with a mean value of 7.28 and least for potato chips with a value of 7.15. Immediately after the intake of beverage/eatables, fruit juice and cold drink showed a sharp decline in pH while other groups showed a sharp increase in the pH level. However, after 30 minutes, all the groups showed a tendency to return toward baseline values. In cold drink, chocolate, cream Biscuit groups, mean pH levels were less than at baseline, while in potato chips and fruit juice, it was almost equal or slightly higher than baseline value. At all subsequent follow-up intervals, a statistically significant intergroup difference was observed (p = 0.001) (Table 3 and Graph 1).

**Table 3:** **Table 3:** Mean pH values in different groups at different time intervals

		*Baseline*				*After exposure*				*5 min*				*10 min*				*15 min*				*30 min*			
*Time interval*		*Mean*		*SD*		*Mean*		*SD*		*Mean*		*SD*		*Mean*		*SD*		*Mean*		*SD*		*Mean*		*SD*	
Group I		7.15		0.15		7.50		0.18		7.29		0.18		7.21		0.18		7.19		0.17		7.17		0.18	
Group II		7.28		0.17		6.33		0.30		6.92		0.16		7.14		0.16		7.22		0.15		7.29		0.18	
Group III		7.20		0.22		6.29		0.37		6.75		0.29		6.58		0.29		6.92		0.22		7.08		0.20	
Group IV		7.26		0.14		7.57		0.10		7.49		0.09		7.25		0.06		7.22		0.06		7.21		0.06	
Group V		7.24		0.17		7.63		0.20		7.41		0.16		7.25		0.14		7.18		0.12		7.13		0.13	
Total		7.23		0.18		7.06		0.66		7.17		0.34		7.09		0.31		7.15		0.19		7.18		0.17	
ANOVA “F” value		4.610				374.802				136.350				119.080				32.532				12.505			
p-value		0.001 (HS)				0.001 (HS)				0.001 (HS)				0.001 (HS)				0.001 (HS)				0.001 (HS)			

SD: Standard deviation; HS: Highly significant

**Table 4:** **Table 4:** Mean buffer capacity of all study subjects

		*Number*		*Mean*		*SD*		*Range*	
Buffer capacity		50		9.34		1.507		4-12	

SD: Standard deviation

### Buffer Capacity

The mean buffer capacity was observed to be 9.34 with a standard deviation of 1.507 and in between the range of 4 and 12 (Table 4). The buffer capacity of the study subjects was based on colorimetric method of measurement and values were interpreted based on the conversion table provided by the manufacturer of the kit used for measurement.

## DISCUSSION

Dental disease is an expensive affair to health care services which includes dental caries, dental erosion, and developmental defects of enamel. However, in modern society, it has been perceived that prevalence of tooth loss by dental caries or dental erosion is rapidly increasing. The dietary components have also been contemplated to be the contributing factor for development of enamel defects.^10^ Relation allying diet and nutrition, and oral health and diseases is a synergistic, i.e., two-way street. Alterations in nutrients consumption secondary to changes in diet have an effect on the integrity of the teeth.^11^ It has been perceived that prolonged and frequent oral exposures to certain types of carbohydrates are very critical to caries activity in humans, animals, and *in vitro.* The bacterial community attached around teeth uses simplified sugars like glucose, fructose, and sucrose in their biochemical glycolytic pathways and produces energy-giving acid as a by-product.

Consequently, the demineralization of tooth by fermentable carbohydrates ensues as acidity of tooth-plaque interface falls to a critical point. The frequency of demin-eralization is reliant on the absolute pH decline, as well as the length of time for which pH is lower than a level that nurture dissolution of enamel.^12^ Although fermentable carbohydrates having dietary origin are the necessary component of dental caries process, the extent of their contribution to the process remains uncertain.

A vast amount of clinical and laboratory-based research studies have been conducted on the effects of carbonated beverages in association with dental caries and erosion,^13^ but very less has been carried out on humans and there is dilemma of data on fruit juices, chocolate, and other most commonly consumed snacks and their acidogenic potential. Hence, a comparative randomized clinical trial was performed to recognize the saliva buffering capacity and salivary pH changes after consumption of potato chips, packaged fruit juice, cold drink, chocolate, and cream biscuits in children.

In the present study, glass combination electrode was used for calculating salivary pH, as it is an accurate, sensitive, and accepted methodology. It is preferred due to its accuracy, as it is said to be unaffected by the varied factors which cause errors.^14^ The salivary pH has individualistic daily, monthly, and annual cycles. The lowest salivary pH was found to be at 9 A.M., and this varied markedly with the subject. It is said to be related to body metabolism, as pH of saliva increases during the day, especially between 9 and 11 A.M. when metabolic activities usually become greater.^15^ For this reason, all the subjects and their parents were instructed that child does not consume anything for up to 2 hours preceding appointment and samples were collected in mornings at 11 **am.**

The present study included five groups: Potato chips; packaged fruit juice, cold drink, chocolate, and cream biscuits. Our study results indicated a drop in salivary pH after consumption of cold drink and packaged fruit juice with further significant drop in pH in cold drink group when compared with packaged fruit juice users. But an increase in pH was seen in subjects immediately after consuming potato chips, chocolate, and cream biscuits. The baseline pH in all the groups varied and ranged from 7.15 to 7.28 with highest being in group II, though when compared, none of the between-group comparisons were significant statistically, except groups I *vs* II and groups I *vs* IV. Maximum difference was observed between groups I and II, i.e., 0.133 and minimum between groups II and IV, i.e., 0.018.

Salivary pH also showed an increase in pH immediately (7.50 ± 0.182) after eating potato chips from baseline pH (7.15 ± 0.154). After 5 minutes, the pH decreased (7.29 ± 0.188) and further reduced after 10 minutes (7.21 ± 0.187) and 15 minutes (7.19 ± 0.51), and reached baseline after 30 minutes, which ranged from 6.26 to 7.38 with a mean value of 7.17 ± 0.189. Pairwise comparisons showed a significant difference between all the time intervals except baseline to 10 minutes, baseline to 15 minutes, baseline to 30-minute intervals, 10 *vs* 15 and 30 minutes, as well as from 15 to 30 minutes respectively.

The result of present study are in concurrence with the study of Saha et al^16^ which was done among the first-year undergraduate dental students in Lucknow, where Guava and apple-based packed juices were used in the study and exhibited pH changes from baseline to 30 minutes ranging from 7.17 to 6.58, 7.08, 7.31, 7.31. Azrak et al^8^ in Germany, in their study including children with a mean age of 4.4 years, showed results similar to our study by observing the pH difference from baseline to 25 minutes (Δ pH = -0.20 at baseline, -0.50 at 5 minutes, -0.24 at 10 minutes, -0.16 after 15 minutes, and -0.01 after 25 minutes bringing back the pH to baseline value) after consuming packaged apple juice. The probable reason for immediate decline in salivary pH in our study could be the intrinsic acidity of packaged fruit juices, rendering it more liable to counter salivary buffers. It has been observed that packaged fruit juices and fruit-based carbonated beverages, having increased buffering capacities, may instigate an elongated drop in oral pH.^17^ Although the quantity of acidic beverages normally ingested by children may be negligible, the presence of inability to clear retentive substrate, immature enamel, and inadequate neuromuscular coordination makes them more prone to dental erosion.

The result of present study reported a decline in pH immediately after consuming carbonated beverage and after 5 minutes, which are similar to the study reports of Lehl et al^18^ (below 5.5), Moazzez et al,^19^ Sanchez et al^20^ (6.70-5.80), and Sardana et al^21^ (6.20-5.30). The carbonated beverages contain citric acid, phosphoric acid, and maleic acid,^10^ which may be a reason for decrease in the salivary pH as observed in the study and enamel demin-eralization. Existence of acids in carbonated beverage would be the cause for immediate decline in salivary pH which may have relinquished the buffering capacity of saliva. In a case-control study, the patients with erosion demonstrated a longer period of time at low pH following drinking of a carbonated drink, while the controls showed longer exposure to low pH on the labial surfaces of the upper incisor.^19^ Titratable acid has been found by several studies to affect the erosive potential of soft drinks.^13^

There was an increase (7.63 ± 0.206) in salivary pH from baseline (7.24 ± 0.173) immediately after consumption of cream biscuits. After 5 minutes, there was a decrease in pH (7.41 ± 0.166) which was sustained for 10-, 15-, and 30-minute time intervals (7.25 ± 0.140, 7.18 ± 0.129, and 7.13 ± 0.137). After 30 minutes, the decrease in pH (7.13 ± 0.137) was observed to be well below the baseline level. On pairwise evaluation, all differences were found to be significant statistically except from baseline to 10 minutes. Maximum mean difference has been observed between immediately after intake and 30 minutes (0.496), while minimum of difference was observed between baseline and 10 minutes after intake of cream biscuits (0.012).

### Between-group Comparison

In our present study, the group III consuming commercially available carbonated cold drink showed maximum drop in salivary pH (6.29), just after consumption when compared with group II packaged fruit juice (6.33), followed by a gradual recovery within 30 minutes of study. This maximum drop could be accredited to the comparatively lower intrinsic pH, sugar content and high titrable acidity of commercially available carbonated soft drink.^9^

However, the present study differs from the studies conducted by Banan and Hegde,^22^ Azrak et al,^8^ and Goel et al,^7^ which concluded that mean pH drop in fruit juice is greater than that observed in carbonated beverage. The ability of fruit juices to resist pH changes may be possibly dependent upon the type of acid present as explained and observed in a study where carbonated beverages contained phosphoric acid as the only acid, while citric acid and ascorbic acid were predominantly present in the fruit juices with presence of high sugar content in fruit juices resulting in high titrable acidity. Astoundingly, a study by West et al has concluded that citric acid causes far more erosive potential than phosphoric acid.^23^

The potato chips, chocolate, and cream biscuits groups (I, IV, and V respectively) showed an increase in salivary pH immediately after consumption when compared with groups II and III. This may be attributed to the acids present in both beverages causing decrease in pH and high content of phosphates and carbonates in the other three groups, leading to increase in buffering capacity. It was shown that the “sour” and “sweet” but also “bitter” and “salty” are stimulants for the salivary flow (Dawes and Watanabe 1987, Edgar 1992).

The flow rate of saliva as well as the buffering capacity vary among different individuals and can eventually create differences in results among other subject groups.^24^ In the present study, buffer capacity of 50 subjects was measured by using colorimetric method by using GC Saliva Check Buffer test strips (GC India dental Pvt. Ltd), as they are usable for buffer capacity detection in dental offices.^25^ The data obtained concluded that the mean buffer capacity was observed to be 9.34 with a standard deviation of 1.507 and in between the range of 4 to 12. The mean salivary buffer capacity obtained in the study can be said to be near or equivalent to normal levels of buffering capacity of saliva according to values interpreted in the conversion table used for colorimetric method measurement. Roos and Donly^24^ showed that plaque pH levels were remarkably less acidic in children aged between 4 and 6 years than in adults aged 16 to 35 years. The study also showed that plaque pH remained below 6.0 for greater interval in adults.^24^

Distinct host factors, such as frequency of consumption and pattern of mastication can contribute to total acidogenic potential. Weatherell et al^24^ showed that the pH value was found to be variable from site to site inside oral cavity. A lower pH was found within plaque on posterior teeth, whereas the anterior teeth showed a higher plaque pH. The mandibular teeth generally possessed a higher pH than maxillary teeth, which was attributed to the rate of oral clearance. The sequence of plaque pH found in that study was also consistent with the data.^24^

In our present study, clinical trial was carried out using only one type, among the various commercially available ready products. However, more studies having much larger sample sizes with above products and individual ingredients of these products with a control group should be conducted *in vitro* as well as *in vivo.* It has been documented that there is marginal pH drop while using straw for consumption, but the children usually have habit of drinking from glass. So it can be said that, beverage intake cannot be standardized, as food preferences differ from individual to individual as well as from society to society.

However, erosion and caries process are as different as their histobiological appearances, but both conditions occurring simultaneously could be detrimental to dental hard tissues. As dental professionals, we need to educate our patients about the repercussions of soft drink consumption and furnish solutions to minimize the risk. The results suggest that the future endeavor needs to be directed toward modifying drinking habits as well as limiting frequency of intake of acidic drinks to avoid erosion.

## CONCLUSION

The conclusions of the present study are:

- It was found that commercially available carbonated cold drink caused greater drop in salivary pH than that of packaged fruit juice immediately after consumption and after 5 minutes.
- Maximum drop seen in cold drink when compared with fruit juice, whereas there was increase in pH immediately after consuming potato chips, chocolate, and cream biscuits. Although after 5 minutes the pH started to fall and reached back to near baseline levels for potato chips at 30-minute interval, the pH fell below the baseline level observed for chocolate and cream biscuits after 30-minute interval.
- The maximum pH increase was observed in cream biscuits with 7.63 ± 0.20.
- The mean salivary buffer capacity was measured to be normal for the study subjects aged between 8 and 12 years.
